# Anxiety and coping in the situation of pandemic as factors of sleep-related complaints during lockdown

**DOI:** 10.1192/j.eurpsy.2021.687

**Published:** 2021-08-13

**Authors:** E. Rasskazova, A. Yavorovskaya

**Affiliations:** 1 Clinical Psychology, Moscow State University, Moscow, Russian Federation; 2 Faculty Of Psychology, Lomonosov Moscow State University, Moscow, Russian Federation

**Keywords:** lockdown, Anxiety, coping, sleep-related complaints

## Abstract

**Introduction:**

Sleep-related complaints are among the most common during pandemic, along with anxiety and depression (Huang, Zhao 2020, Rajkumar 2020). Their prevalence is associated with anxiety about the pandemic (Roy et al., 2020), online information search (Moghanibashi-Mansourieh, 2020, Wang et al., 2020).

**Objectives:**

The aim was to reveal relationship between the type anxiety and coping during pandemic and sleep-related complaints after 3-4 weeks of lockdown.

**Methods:**

203 adults aged 18 to 59 years filled situational version COPE (Carver et al., 1989) and scales measuring anxiety of infection and pandemic consequences (Tkhostov, Rasskazova, 2020) in the mid-April 2020 after 2-3 weeks of lockdown in Russia. After 3-4 weeks, they filled in a modified insomnia severity index (Morin, 1991) appraising how much worse their sleep and daytime functioning during this period (Cronbach’s alpha .62-.73).

**Results:**

Prevalence of sleep and day functioning related complaints during lockdown varied 19.3%-30.5%. Complaints were associated with anxiety only if it reaches dysfunctional level (interfering with daily activities, r = .17-.34, p <.05) and coping strategy of mental disengagement (r = .15-.19, p <.05). Sleep complaints were related to substance use to cope with lockdown problems while complaints on daytime functioning correlated were more common among young respondents (r = -0.22, p <0.01).

**Conclusions:**

Complaints about poor sleep during a pandemic are not related to the general severity of pandemic anxiety, but to the dysfunctional level of anxiety and attempts to avoid it. Research is supported by the Russian Foundation for Basic Research, project No. 20-013-00740.

**Conflict of interest:**

Research is supported by the Russian Foundation for Basic Research, project No. 20-013-00740.

